# Application of the Deep Neural Network in Retrieving the Atmospheric Temperature and Humidity Profiles from the Microwave Humidity and Temperature Sounder Onboard the Feng-Yun-3 Satellite

**DOI:** 10.3390/s21144673

**Published:** 2021-07-08

**Authors:** Qiurui He, Zhenzhan Wang, Jiaoyang Li

**Affiliations:** 1 School of Information Technology, Luoyang Normal University, Luoyang 471934, China; heqiurui@lynu.edu.cn; 2 Key Laboratory of Microwave Remote Sensing, National Space Science Center, Chinese Academy of Sciences, Beijing 100190, China; 3 Department of Electrical and Computer Engineering, Michigan State University, East Lansing, MI 48824, USA; lijiao@msu.edu

**Keywords:** SNN, DNN, MWHTS, FY-3, atmospheric temperature and humidity profiles, bias correction, retrieval scheme of atmospheric parameters

## Abstract

The shallow neural network (SNN) is a popular algorithm in atmospheric parameters retrieval from microwave remote sensing. However, the deep neural network (DNN) has a stronger nonlinear mapping capability compared to SNN and has great potential for applications in microwave remote sensing. The Microwave Humidity and Temperature Sounder (Beijing, China, MWHTS) onboard the Fengyun-3 (FY-3) satellite has the ability to independently retrieve atmospheric temperature and humidity profiles. A study on the application of DNN in retrieving atmospheric temperature and humidity profiles from MWHTS was carried out. Three retrieval schemes of atmospheric parameters in microwave remote sensing based on DNN were performed in the study of bias correction of MWHTS observation and the retrieval of the atmospheric temperature and humidity profiles using MWHTS observations. The experimental results show that, compared with SNN, DNN can obtain better bias-correction results when applied to MWHTS observation, and can obtain higher retrieval accuracy of temperature and humidity profiles in all three retrieval schemes. Meanwhile, DNN shows higher stability than SNN when applied to the retrieval of temperature and humidity profiles. The comparative study of DNN and SNN applied in different atmospheric parameter retrieval schemes shows that DNN has a more superior performance.

## 1. Introduction

As the basic parameters of the atmosphere, temperature and humidity profiles play an important role in the research and applications of atmospheric science, such as numerical weather forecast, climate change research, and strong convective weather forecast and analysis [1,2,3,4]. Global reanalysis datasets developed by the European Centre for Medium-Range Weather Forecasts (ECMWF) or the National Centers for Environmental Prediction (NCEP) are generated by the assimilation system that assimilates a lot of information from satellites’ and radiosondes’ data. The global reanalysis datasets are good estimations of the state of the atmosphere and can be used as a reference in the retrieval of the atmospheric parameters and the climate change research. Although the global reanalysis can provide a variety of atmospheric parameters with high spatial resolution and high accuracy, it suffers from a long time delay (one month or more) compared with the satellite observations, which cannot meet the requirements for real-time in atmospheric applications, such as numerical weather forecasting, extreme weather monitoring, etc. Microwave radiometer, which takes a passive microwave remote sensing approach, is an important instrument to monitor the Earth–Atmosphere system, and its observation is an important data source to obtain information about atmospheric temperature and humidity in atmospheric science [5,6]. The retrieval algorithm can be used to convert microwave remote sensing measurements into atmospheric temperature and humidity parameters [7].

The atmospheric temperature and humidity profile retrieval algorithms based on passive microwave observation have been developed for more than 60 years, and the retrieval algorithms can be summarized into two categories: physical retrieval algorithms and statistical retrieval algorithms [1,8]. The essence of the physical retrieval algorithm is to estimate the atmospheric temperature and humidity profiles by inverting the radiative transfer equation, which is an ill-posed problem and usually requires a priori information to constrain the equation to obtain a unique solution (i.e., the temperature and humidity retrievals) [9,10,11,12]. The essence of the statistical retrieval algorithm is to estimate the atmospheric temperature and humidity profiles based on the statistical relationship between the atmospheric temperature and humidity parameters and the microwave observations, without involving any physical concepts [13,14,15,16]. In addition, an algorithm has also been classified as a physical retrieval algorithm if a physical model is used in retrieving, and as a statistical inversion algorithm otherwise.

Among the many retrieval schemes of atmospheric temperature and humidity profiles using the passive microwave observations, three neural-network-based schemes have been successfully and widely applied for most of the atmospheric scenes, especially the clear sky scene [17,18]. The first retrieval scheme is based on the one-dimensional variational algorithm (1DVAR), which is a typical representative of physical retrieval algorithms. It adjusts the initial value of the atmospheric parameter through an iterative process, with the aim that the bias between the observed brightness temperature and the simulated brightness temperature calculated by the radiative transfer model using the initial value (observation bias) satisfies a certain threshold, at which point the adjusted initial value is the retrieved value of the atmospheric parameter [19]. However, the 1DVAR requires the observation bias to satisfy the unbiased and Gaussian characteristics, so the observation bias must be quantified and removed, and the removal of this bias can be performed by using a bias-correction method based on neural networks (NNs) [20,21]. The second retrieval scheme is based on the statistical relationship between the observed brightness temperature and the atmospheric temperature and humidity profiles. NNs can be applied to build the statistical model between the observed brightness temperature and the temperature and humidity profiles [22,23,24]. The third retrieval scheme, which is based on the statistical model between the simulated brightness temperature calculated by the radiative transfer model and the atmospheric temperature and humidity profiles, is similar to the second retrieval scheme. However, compared to the second retrieval scheme, the observed brightness temperature must be corrected before retrieving; that is, the bias between the simulated brightness temperature and the observed brightness temperature must be reduced as much as possible (bias correction). Therefore, in the third retrieval scheme, NNs can be both applied to the bias correction and the statistical modeling of the simulated brightness temperature and the temperature and humidity profiles. In addition, the third retrieval scheme can be classified as a physical retrieval scheme due to the calculation of the simulated brightness temperature are performed by the radiative transfer model [17].

At present, although NNs are widely used to retrieve atmospheric temperature and humidity profiles using passive microwave observations, it is almost the SNNs that are extensively used, and they usually contain a hidden layer with a small number of neurons that are based on the error backward propagation algorithm. Each neuron in the hidden layer performs a nonlinear computation, and the small number of neurons in the hidden layer limits the nonlinear mapping capability of SNN. When there are thick clouds or/and rain in the sight of the microwave radiometer, the nonlinear relationship between the measurements of the microwave radiometer and the atmospheric temperature and humidity profiles is complicated, and SNN may not be able to accurately model the nonlinearity between the input and output samples, which leads to the poor accuracy or even failure of the retrieval of the temperature and humidity profiles based on SNN. However, compared with SNN, the nonlinear mapping capability of DNN has been greatly improved [25,26]. Therefore, DNN has great potential for applications in retrieving atmospheric parameters using passive microwave observations.

MWHTS is a key payload onboard the new generation of polar-orbiting meteorological satellites Fengyun-3C (FY-3C, Beijing, China) and FY-3D. It is a microwave radiometer that integrates humidity and temperature sounding to detect atmospheric humidity and temperature profiles simultaneously. In this paper, a study on the applications of DNN in three retrieval schemes for temperature and humidity profiles using MWHTS measurements is recounted. The retrieval results are analyzed and compared with those of SNN, with the aim of exploring the potential of DNN in retrieving atmospheric parameters by using passive microwave observations. This paper is organized as follows: Section 2 describes the data and model used in this study. In Section 3, the retrieval algorithm and experimental design are presented, mainly including the introduction of DNN and the description of application methods of DNN in three retrieval schemes. Section 4 presents the experimental results and analysis. Section 5 presents the conclusions.

## 2. Data and Model

### 2.1. MWHTS Characteristics

As a total power microwave radiometer, MWHTS is an important payload onboard FY-3C and FY-3D satellites, which were launched in September 2013 and November 2017, respectively, and both payloads are operating normally in orbit and have accumulated rich atmospheric sounding data. MWHTS performs the cross-track scanning along the orbit with the angle of ±53.35° from the nadir to inspect 98 nominal fields of view (FOVs) in each scan line, which is corresponding to the scanning of the swath of 2645 km in 2.667 s. MWHTS has eight temperature sounding channels with frequencies near the 118.75 GHz oxygen absorption line for sounding temperature from the surface to the upper atmosphere and five humidity sounding channels with frequencies around the 183.31 GHz water vapor absorption line for sounding water vapor and precipitation from the surface to about 300 hPa, and two window channels with frequencies at 89.0 and 150.0 GHz, respectively, for providing information about the surface parameters [27]. Table 1 lists major channel characteristics of MWHTS onboard Fengyun-3C and -3D satellites.

### 2.2. Data and Model

The datasets used in this study included the following: (1) Level 1b brightness temperatures of MWHTS onboard FY-3D from the National Satellite Meteorological Center (NSMC) (http://satellite.nsmc.org.cn accessed on 15 November 2019)—the quality of MWHTS observations at ECMWF has been evaluated, and the detailed description of the evaluated results can see Lawrence et al. [28]; and (2) European Centre for Medium Range Weather Forecasts (ECMWF) ERA-Interim reanalysis dataset obtained from the ECMWF website (http://apps.ecmwf.int/datasets accessed on 15 November 2019). Many global atmospheric sounding measurements are assimilated with the 12-h analysis window by using four-dimensional variational analysis to produce ERA-Interim [29,30]. In this study, the profile parameters and the surface parameters from ERA-Interim were used to build the atmospheric parameter dataset, where the profile parameters include: temperature, specific humidity, and specific cloud liquid content, which have a total of 37 pressure levels unevenly from 1000 to 1 hPa, and the surface parameters include the following: 2 m temperature, 2 m dewpoint temperature, surface pressure, skin temperature, and 10 m u wind component and 10 m v wind component. The parameters used to build the atmospheric parameter dataset were with a horizontal resolution of 0.5 × 0.5° and a temporal resolution of 6 h. The data from the ocean area of 25° N–45° N and 160° E–220° E from 1 September 2018 to 31 August 2019 were selected to build the atmospheric parameter dataset and MWHTS brightness temperature dataset.

In the three retrieval schemes used in this study, it was necessary to calculate the simulations of MWHTS, especially for the retrieval scheme based on the physical retrieval algorithm, where the radiative transfer model is an essential part. In this study, the fast radiative transfer model RTTOV (Radiative Transfer for Television and Infrared Observation Satellite Operational Vertical Sounder, Washington, DC, USA), Version 11.3, developed by ECMWF, was used to calculate MWHTS simulated brightness temperatures. RTTOV can calculate simulated brightness temperatures for a wide range of satellite-based microwave radiometers, with an accuracy that can meet the application requirements [31]. The emission-based RTTOV mode was performed to simulate the brightness temperature of MWHTS due to the lack of information about ice clouds and rain.

### 2.3. Data Preprocessing

**Figure 1:**
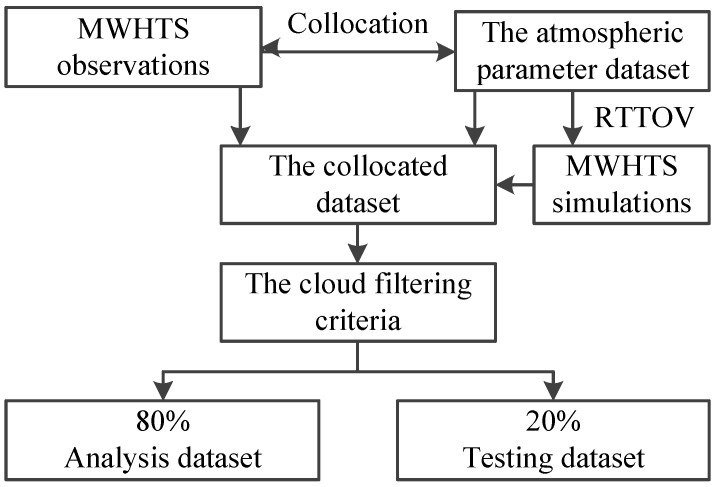
The schematic of the data-preprocessing procedure.

According to the purpose of this paper, for applying the data used in this study in the three retrieval schemes, the data-preprocessing process was as follows. First, building the collocated dataset. As for the criteria of the collocation between the MWHTS observed brightness temperatures and the atmospheric parameter dataset, the time and absolute distance in latitude and longitude are less than 0.5 h and 0.1°, respectively. Second, calculating the simulated brightness temperatures of MWHTS in the collocated dataset in which the profile parameters and the surface parameters are input to RTTOV to calculate the simulations. Then, after filtering out the scattering-affected data in the collocated dataset, we took the vertical integral cloud liquid water of 0.05 mm as the threshold of precipitation. If the integral cloud liquid water in one group of collocated dataset exceeds 0.05 mm, then that group’s data are considered as scattering-affected data and are removed. Finally, 80% of the collocated dataset after filtering out the scattering-affected data containing the observed brightness temperatures, the simulated brightness temperatures, and the atmospheric parameters is randomly selected as the analysis dataset with 848129 collocated samples, and the remaining 20% are set as the testing dataset with 212033 collocated samples. The overall data preprocessing procedures are summarized in Figure 1.

## 3. Algorithm and Experiment Design

### 3.1. Deep Neural Network

In 2006, deep learning was introduced in the research of Hinton and his students, which initiated the research wave of multilayer neural networks, and many scholars began to study the applications of DNN in various fields [32]. In short, DNN can be understood as a neural network that contains many hidden layers and the hidden layers contain many neurons. DNN has a similar network structure to SNN widely used in atmospheric parameters retrieval, i.e., it contains an input layer, several hidden layers, and an output layer. Moreover, as with SNN, DNN can be based on the backpropagation learning algorithm, which targets the minimum squared error of prediction and adjusts the threshold and weight of the network to close the expected value. SNN and DNN are both fully connected networks, where each neuron in each layer is connected to all neurons in the next layer. In the hidden layer, each neuron performs nonlinear computation on all input vectors to achieve a nonlinear description of the relationship between input and output samples, which makes them have nonlinear mapping capability [33]. Building a DNN or SNN will involve error backpropagation algorithms, loss functions, and gradient descent algorithms, etc., as detailed in References [26,34,35]. In addition, the structural similarity between DNN and SNN makes them operate in similar ways when applied to microwave remote sensing atmospheric parameters, such as the building of learning samples, the application of error backpropagation algorithm, the setting of activation function, etc. However, the differences in the number of hidden layers and the number of neurons in each hidden layer between DNN and SNN make DNN have a stronger learning ability than SNN and, therefore, show a superior application.

The purpose of DNN, as a type of neural network, is to build a statistical model between input and output samples through supervised learning in a training dataset, and to make predictions on new input samples from the validation dataset when they are fed into the established statistical model. In this study, the application of DNN to the retrieval of atmospheric temperature and humidity profiles using MWHTS observations involved two main aspects: the building of the DNN-based observation-bias-correction model and the building of the DNN-based retrieval model for temperature and humidity profile.

**Figure 2:**
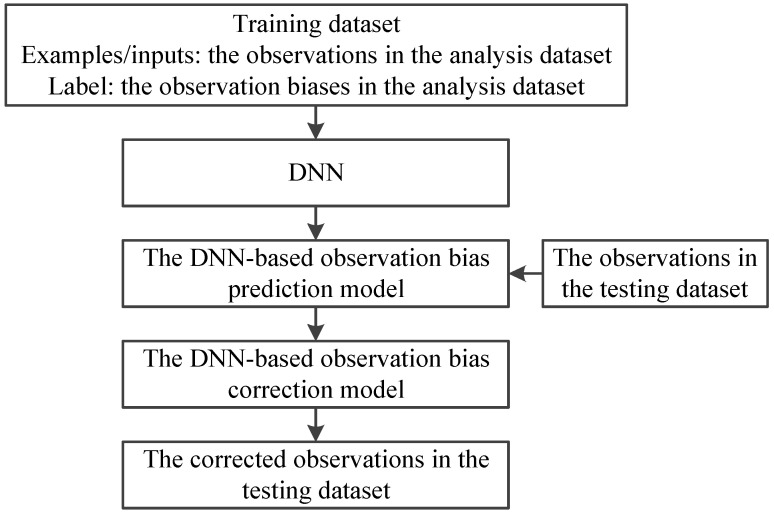
The schematic of the observation-bias correction.

For MWHTS-observation-bias correction, DNN was used to build a statistical model between MWHTS-observed brightness temperatures and MWHTS observation biases for predicting the observation biases, and the observation biases are defined as follows:

(1) $${\tilde{R}}_{B}=\tilde{R}-{\tilde{R}}_{S}$$

where $\tilde{R}$ is the observed brightness temperatures, and ${\tilde{R}}_{S}$ is the simulated brightness temperatures. The predictions of the observation bias are obtained as follows. First, the training dataset of DNN is established, i.e., the MWHTS observed brightness temperatures in the analysis dataset are set as the input samples, and the observation biases in the analysis dataset are set as the output samples. Then, the four-layer network structure of DNN (i.e., one input layer, two hidden layers, and one output layer) is built. The training dataset is used to train the DNN model, and the DNN-based observation-bias prediction model is built. Finally, the MWHTS observed brightness temperatures from the testing dataset are fed into the DNN-based observation-bias-prediction model to obtain the predictions of the observation bias, and the observation biases in the testing dataset are used to verify the correction effect of the DNN-based observation-bias-prediction model. Further details of the training and testing of the DNN-based observation-bias-prediction model are contained in Section 3.3 and Section 4, respectively. Based on the predictions of the observation bias, the observation-bias-correction model and the corrected brightness temperatures are obtained:

(2) $${\tilde{R}}_{C}=\tilde{R}-{\tilde{R}}_{B}^{\prime }$$

where ${\tilde{R}}_{B}^{\prime }$ is the prediction of the observation bias. An illustration of the schematic of the observation-bias-correction process is displayed in Figure 2.

**Figure 3:**
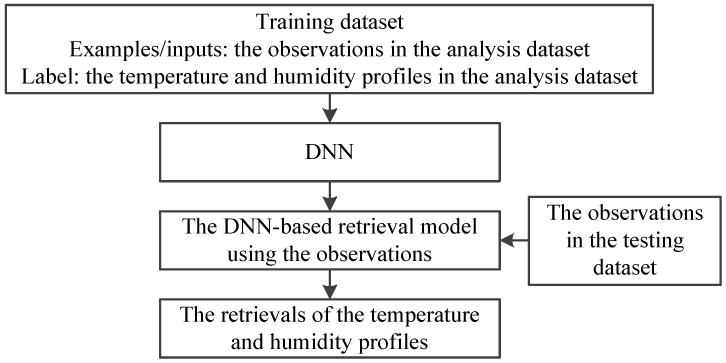
The schematic of the retrieval based on the DNN-based retrieval model using the observations.

**Figure 4:**
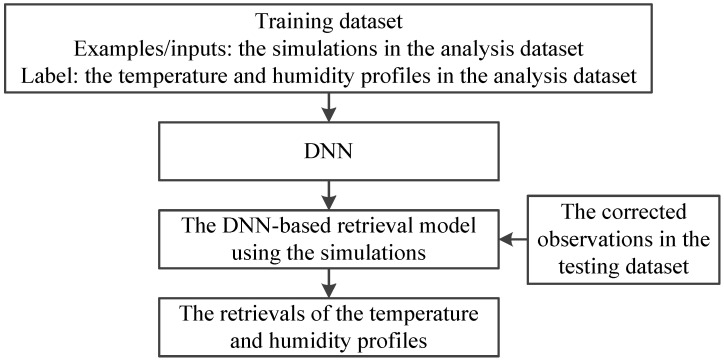
The schematic of the retrieval based on the DNN-based retrieval model using the simulations.

For the retrieval of atmospheric temperature and humidity profiles, both MWHTS observed brightness temperature and MWHTS simulated brightness temperature can be used to build the retrieval model. The DNN-based retrieval model using the observed brightness temperature is built as follows. The training dataset of DNN is established, i.e., MWHTS observed brightness temperatures in the analysis dataset are set as the input samples, and the atmospheric temperature and humidity profiles in the analysis dataset are set as the output samples. Then, the four-layer network structure (i.e., one input layer, two hidden layers, and one output layer) of DNN is built, and DNN is trained by using the training dataset. Thus, the DNN-based retrieval model, using the observations, is built. If DNN is trained with the simulated brightness temperature instead of the observed brightness temperature in the training dataset, the DNN-based retrieval model using the simulations is obtained. Finally, the observed brightness temperatures from the testing dataset are fed into the DNN-based retrieval model using the observation or the corrected brightness temperature from the testing dataset are fed into the DNN-based retrieval model, using the simulations to obtain the retrievals of the temperature and humidity profiles, and the temperature and humidity profiles in the testing dataset are used to verify the retrieval of the temperature and humidity profiles. Further details of the training and testing of the DNN-based retrieval models are also contained in Section 3.3 and Section 4, respectively. The schematic of the retrieval of the atmospheric temperature and humidity profiles using the above two DNN-based retrieval models are summarized in Figure 3 and Figure 4, respectively.

### 3.2. The 1DVAR Algorithm

The 1DVAR algorithm is generally labeled under the general term of physical retrieval that inputs the initial values of atmospheric parameters to the radiative transfer model and adjusts the initial values through an iterative process with the aim of fitting the simulations from the radiative transfer model to the observations from the satellite. The 1DVAR algorithm mainly includes two parts: one is the radiative transfer model for the simulations of brightness temperature; the other is the minimization of the cost function. Assuming that the errors in the observations are neither biased nor correlated, Gaussian distribution, the optimal estimate of the atmospheric state variable, S, can be obtained by minimizing the following cost function [36]:

(3) $$\;\;\;\xi \;\;\;=\frac{1}{2}{\left(S-S_{a}\right)}^{T}C_{SS}^{-1}\left(S-S_{a}\right)+\frac{1}{2}{\left[f\left(S\right)-\tilde{R}\right]}^{T}C_{\Psi \Psi }^{-1}\left[f\left(S\right)-\tilde{R}\right]$$

where $C_{\Psi \Psi }$ is the observation-error covariance matrix, which is the sum of the covariance error in the brightness temperature simulations and the sensor noise; $C_{SS}$ is the background covariance matrix; $S_{a}$ is the background state variable; f is the forward operator that simulates the satellite observations at the atmospheric state variable, ***S***; and T represents the matrix transpose. By minimizing the cost function, ξ, the optimal solution is obtained as follows:

(4) $$S_{n+1}=S_{a}+C_{SS}K_{n}^{T}{\left[K_{n}C_{SS}K_{n}^{T}+C_{\Psi \Psi }\right]}^{-1}\left[\tilde{R}-f\left(S\right)-K_{n}\left(S_{a}-S_{n}\right)\right]$$

where ***K*** is the tangent linear function of f at point ***S***, *n* is the iteration index, and ***S***_1_ is the initial state variable.

**Figure 5:**
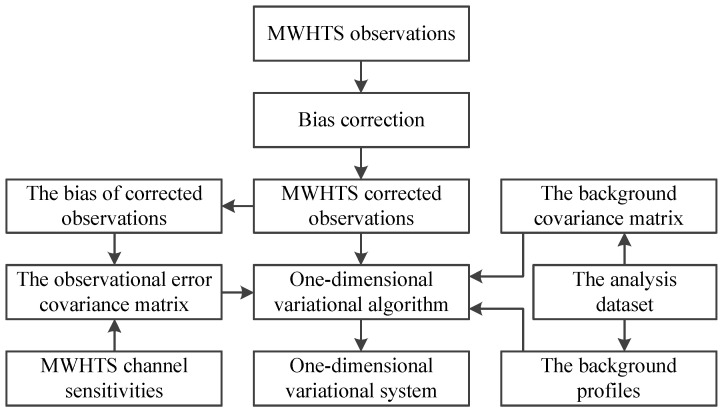
The schematic of building the MWHTS 1DVAR retrieval system.

In this study, the parameters of the 1DVAR for retrieving atmospheric temperature and humidity profiles using MWHTS observations were specifically set as follows. The averages of the temperature and humidity profiles in the analysis dataset were taken as both the background state variable, ***S_a_***, and the initial state variable, $S_{1}$. MWHTS observation bias was corrected by using DNN, as detailed in Section 3.1, above. After removing biases in the observations, the biases between the observations and the simulations and the sensitivities of MWHTS measured in flight (see Table 1), which are often considered as the instrument channel noise, were used to compute the observation-error covariance matrix, $C_{\Psi \Psi }$; the atmospheric temperature and humidity profiles in the analysis dataset were used to generate the background covariance matrix, $C_{SS}$. For details on the calculation of the observation-error covariance matrix and the background covariance matrix, see References [20,37,38]. The building procedure of the MWHTS 1DVAR retrieval system by setting the parameters of 1DVAR is shown in Figure 5.

### 3.3. Design of Retrieval Experiment

In this study, three commonly used retrieval schemes were designed for retrieving atmospheric temperature and humidity profiles by using MWHTS brightness temperatures to study the effect of DNN on the retrieval accuracy of temperature and humidity profiles in different retrieval schemes. In order to compare the performance of DNN in retrieving temperature and humidity profiles, studies of SNN in the three retrieval schemes were carried out. The three retrieval schemes for retrieving atmospheric temperature and humidity profiles using MWHTS brightness temperatures were specifically designed as follows.

**Figure 6:**
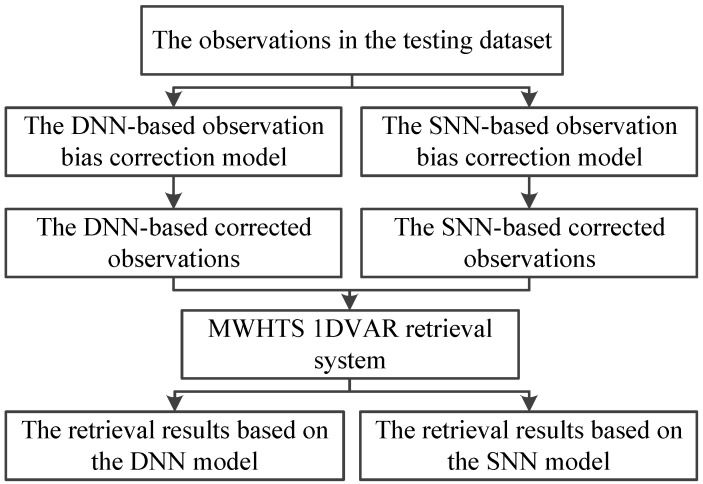
The schematic of the 1DVAR retrieval.

The first retrieval scheme: The 1DVAR retrieval retrieved the atmospheric temperature and humidity profiles based on the MWHTS 1DVAR retrieval system. First, according to the description in Section 3.1, the DNN-based and the SNN-based observation-bias-correction models were built, and they were used to correct MWHTS observed brightness temperatures in the testing dataset, respectively. Then the DNN-based corrected brightness temperatures and the SNN-based corrected brightness temperatures were obtained. Then the parameters of the 1DVAR were set according to the description in Section 3.2, and the MWHTS 1DVAR retrieval system was established. Finally, the DNN-based corrected brightness temperatures and the SNN-based corrected brightness temperatures were input to the MWHTS 1DVAR retrieval system, and the retrieval results of the atmospheric temperature and humidity profiles based on DNN and SNN models were obtained, respectively. The schematic of the 1DVAR retrieval scheme is summarized in Figure 6.

The second retrieval scheme: The NN-based retrieval using the observations retrieved the atmospheric temperature and humidity profiles by the NN-based retrieval model using the observations. The DNN-based retrieval model using the observations and the SNN-based retrieval model using the observations were established, respectively, as described in Section 3.1. Then the MWHTS observed brightness temperatures in the testing dataset were input to the DNN-based retrieval model using the observations and the SNN-based retrieval models using the observations, respectively. Then, the retrieval results of atmospheric temperature and humidity profiles based on DNN and SNN models were obtained, respectively.

The third retrieval scheme: The NN-based retrieval using the simulations retrieved the atmospheric temperature and humidity profiles by the NN-based retrieval model using the simulation. The DNN-based retrieval model using the simulations and the SNN-based retrieval model using the simulations were established, respectively, as described in Section 3.1. Then, the DNN-based corrected brightness temperature and the SNN-based corrected brightness temperature obtained in the first retrieval scheme were input to the DNN-based and SNN-based retrieval models using the simulations, respectively. Then the retrieval results of atmospheric temperature and humidity profiles based on DNN and SNN models were obtained, respectively.

In this study, the DNN and the SNN design used in the three retrieval schemes both produced the best results in terms of reproducing the observations bias and the atmospheric temperature and humidity profiles. The input layers of DNN and SNN used in the observation-bias correction have 15 neurons, which receive the observations of 15 channels of MWHTS, and the output layers have 15 neurons, which output the observation biases corresponding to the observations in the input layers. In the retrievals of the temperature and humidity profiles, the input layers of DNN and SNN also have 15 neurons, which receive the simulations or the observations of MWHTS; the output layers have 74 neurons, which output the temperature and humidity profiles.

For the hidden layers of DNN and SNN applied in the three retrieval schemes, the configurations of neurons, layers, and activation function were determined by extensive testing (CPU: Intel I5, 1.8 GHz; Memory: 16 GB; GPU: NVIDIA GeForce GTX 1060 6 GB). In the extensive testing, the trained DNN and SNN for the observation-bias correction were evaluated by the Root Mean Square Error (RMSE) between the predictions of the observation bias and the observation biases in the testing dataset, and the trained DNN and SNN for the retrieval of the temperature and humidity profiles were evaluated by the RMSE between the retrievals and the temperature and humidity profiles in the testing dataset.

In the three retrieval systems, one hidden layer for SNN and two hidden layers for DNN can ensure adequate training of the model in the extensive testing. Then the Rectified Linear Unit (ReLU) was selected as the activation function in this study because it can overcome the problems of saturation and vanishing gradients [26]. Moreover, compared with Leaky ReLU and sigmoid, DNN or SNN with ReLU can obtain the highest prediction accuracies in the three retrieval schemes. SNN was trained with a different number of neurons in the hidden layer, and the number of neurons increases from 5 to 50, one by one. However, DNN was trained with the number of neurons in the hidden layers; the number of neurons increases from 10 to 1000 in steps of 10, and the same number of neurons was used in both hidden layers. It can be found that small differences in the number of neurons in the hidden layer have a significant impact on the prediction accuracy of the SNN, while the impact on the DNN is small. Targeting the prediction accuracy of the neural network, the number of the hidden layer, the number of neurons in the hidden layers, and the activation function were determined by extensive testing for the three retrieval schemes listed in Table 2 and Table 3. In addition to that, it is important to avoid overfitting in the training. Early stopping can terminate training before overfitting occurs, which split the training dataset and use a subset (20%) as a validation dataset to monitor the performance of NN in the training. An arbitrary maximum number of training epochs is specified, and the training will be terminated if the loss on the validation dataset does not change over a given number of epochs (i.e., patience). The maximum numbers of epochs and the patience for DNN and SNN used in the three retrieval schemes were 2000 and 100, respectively.

## 4. Experimental Results

This section presents the experimental results of DNN and SNN models used to correct the MWHTS observation bias, as well as the retrieval results of atmospheric temperature and humidity profiles when DNN and SNN models are applied to the three retrieval schemes described in Section 3.3, and also presents the experimental results of the stability test of DNN and SNN models in retrieving the atmospheric temperature and humidity profiles.

### 4.1. Bias-Correction Results

**Figure 7:**
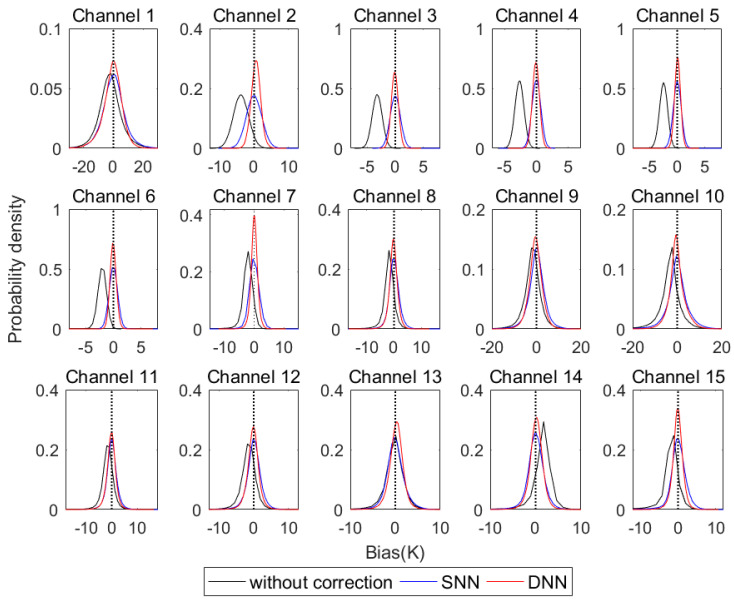
Probability density distribution of observation bias before and after bias correction.

According to the experimental design in Section 3.3, the DNN-based and the SNN-based observation-bias-correction models can be built to correct the MWHTS observation bias in the testing dataset, and then to obtain the DNN-based corrected brightness temperature and the SNN-based corrected brightness temperature, respectively. The comparisons of the probability density distribution of the observation bias for each channel of MWHTS before and after the bias correction are shown in Figure 7.

**Figure 8:**
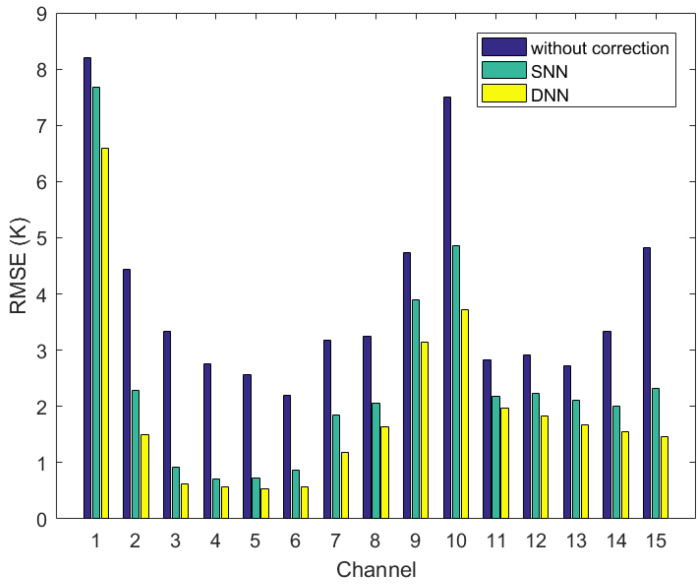
RMSEs between the observed brightness temperatures and the simulated brightness temperatures before and after bias correction.

As can be seen in Figure 7, before bias correction, the observation bias is large in each channel, and after correcting by DNN and SNN, respectively, the observation bias is significantly reduced. Comparing the DNN-based and SNN-based observation-bias-correction model, the DNN-based bias-correction results are significantly better than the SNN-based bias-correction results, and the observation bias corrected by DNN is more consistent with the unbiased and Gaussian properties of the observed brightness temperature required by the 1DVAR algorithm. In order to compare the bias-correction performance of DNN and SNN in each channel of MWHTS in more detail and quantitatively, the RMSE between the observed brightness temperature and the simulated brightness temperature before and after the bias correction is calculated, as shown in Figure 8.

As can be seen from Figure 8, both DNN and SNN can effectively correct the observation bias of MWHTS. For MWHTS window channels 1 and 10 and the channel 9 with peak WF height close to the surface, the observation biases before bias correction are large due to the fact that the surface parameters, the calculation accuracy of the surface emissivity, and the cloud-water parameters in the detection path can enhance the nonlinearity of microwave radiative transfer, which in turn adversely affects the microwave radiation measurements in these channels, and therefore, this may be the main reason why both the DNN-based and the SNN-based observation-bias-correction models are not significant in these channels above. For temperature sounding channels 2–8, since these channels mainly detect the temperature information of the upper atmosphere, the nonlinearity of microwave radiative transfer is relatively weak, and the bias-correction effects of both DNN and SNN models are relatively obvious. In particular, the corrected observation biases of channels 3–6 are less than 0.7 K, and the bias-correction magnitudes are up to 3K. For the humidity sounding channels 11–15, the microwave radiation measured by these channels mainly comes from the water vapor parameter, which has obvious spatial and temporal variation characteristics, and the matching error between the observed brightness temperature and the simulated brightness temperature in the training dataset of the neural networks also has an impact on the bias-correction results. Among them, the bias-correction effect of channel 15 is the most significant, and the bias-correction amplitude can reach 2.5 K. Comparing DNN and SNN in the bias correction, DNN shows superior bias-correction performance in all channels of MWHTS, especially in window channels 1 and 10 and channels 9 and 15, whose peak WF heights are close to the surface, which have stronger nonlinearities in microwave radiation transmission. This verifies that DNN has a stronger nonlinear mapping capability in MWHTS-observation-bias correction than SNN. In a word, DNN has more significant bias-correction performance than SNN when applied to MWHTS-observation-bias correction. However, the retrieval performance of the DNN-based corrected brightness temperatures and the SNN-based corrected brightness temperatures in retrieving atmospheric temperature and humidity profiles needs to be further verified in the first retrieval scheme and the third retrieval scheme.

### 4.2. Results of Retrieval Experiment

This section presents the retrieval results of the temperature and humidity profiles of the three retrieval schemes as designed in Section 3.3. The retrieval accuracies of the three retrieval schemes are verified and analyzed by using the temperature and humidity profiles in the testing dataset, which, from ERA-Interim, are used as the truth values. Based on the distribution characteristics of peak WF heights of MWHTS channels, the retrievals at levels from 1000 to 30 hPa for temperature and from 1000 to 250 hPa for relative humidity are validated, respectively. RMSE between the retrievals and the atmospheric temperature and humidity profiles from ERA-Interim is considered as the standard quantification to validate the retrievals.

#### 4.2.1. The Retrieval Results of the 1DVAR Retrieval

**Figure 9:**
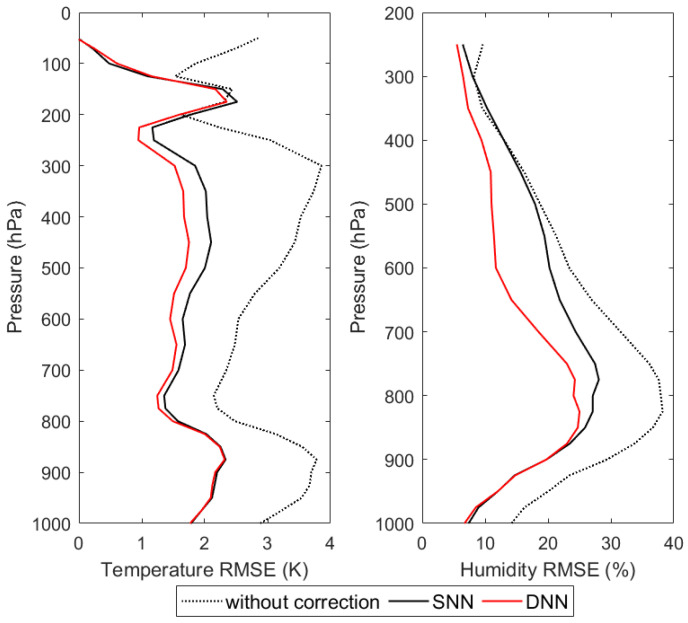
The retrieval RMSEs of the 1DVAR retrieval with respect to ERA-Interim.

According to the experimental design of the first retrieval scheme, the MWHTS observed brightness temperature in the testing dataset, the DNN-based corrected brightness temperature, and the SNN-based corrected brightness temperature obtained in Section 4.1 are respectively input to the MWHTS 1DVAR retrieval system to retrieve the atmospheric temperature and humidity profiles. The comparisons of the retrieval accuracies of the three brightness temperatures are shown in Figure 9, and are also concluded in Table 4, which are given at five different atmospheric levels corresponding to 100, 300, 500, 800, and 950 hPa for temperature and four levels for humidity since the 100 hPa levels are not reliable.

As can be seen from Figure 9, the bias correction of the observed brightness temperature provides a significant improvement in the retrieval accuracy of the temperature and humidity profiles, up to about 2 K at 300 hPa for the temperature retrievals and up to about 11% at 800 hPa for the relative humidity retrieval. It can be seen that the bias correction of observation bias is crucial for the retrieval accuracy of the MWHTS 1DVAR retrieval system. Comparing the performances of the DNN-based and SNN-based observation-bias-correction models in the MWHTS 1DVAR retrieval system, for the temperature profile retrieval, the retrieval accuracies of the DNN-based corrected brightness temperature and the SNN-based corrected brightness temperature are comparable for the upper atmosphere above 200 hPa and the bottom atmosphere from 800 to 1000 hPa. While between 250 and 800 hPa, the retrieval accuracy of the DNN-based corrected brightness temperature is significantly higher than that of the SNN-based corrected brightness temperature, and the retrieval accuracy can be improved by 0.4 K at 450 hPa. For the humidity profile retrieval, the retrieval accuracy of the DNN-based corrected brightness temperature is better than that of the SNN-based corrected brightness temperature in the range of 250 to 850 hPa, especially at 600 hPa, where the retrieval accuracy can be improved by 8.5%. Comparing the performance of the two observation-bias-correction models in the MWHTS 1DVAR retrieval system, it can be found that the DNN-based corrected brightness temperature can obtain higher retrieval accuracies of temperature and humidity profiles.

#### 4.2.2. The Retrieval Results of the NN-Based Retrieval Using the Observations

**Figure 10:**
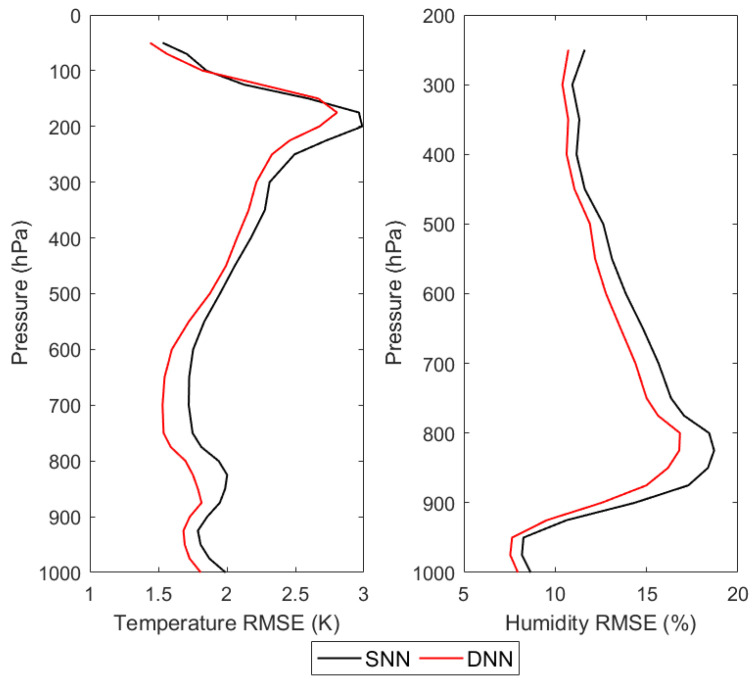
The retrieval RMSEs of the NN-based retrievals using the observations with respect to ERA-Interim.

According to the experimental design of the second retrieval scheme, the observed brightness temperature in the testing dataset is input to the DNN-based and SNN-based retrieval models using the observations, respectively. The output results are the retrieval results of temperature and humidity profiles, and the retrieval accuracies are verified as shown in Figure 10 and Table 5.

From Figure 10, it can be seen that for temperature profile retrieval, the DNN and SNN can obtain comparable retrieval accuracies in the upper atmosphere above 200 hPa, but in the range of 200–1000 hPa, DNN can obtain higher retrieval accuracy, and the maximum improvement is about 0.3 K at 700 hPa compared with SNN. For humidity profile retrieval, the retrieval accuracy of DNN is higher than that of SNN at all atmospheric levels, with a maximum improvement of about 2.5% at 850 hPa compared to SNN. The comparison of the retrieval results of MWHTS observations based on DNN and SNN shows that DNN can obtain higher retrieval accuracies of atmospheric temperature and humidity profiles than SNN in the study of retrieving the temperature and humidity profiles using MWHTS observations.

#### 4.2.3. The Retrieval Results of the NN-Based Retrieval Using the Simulations

**Figure 11:**
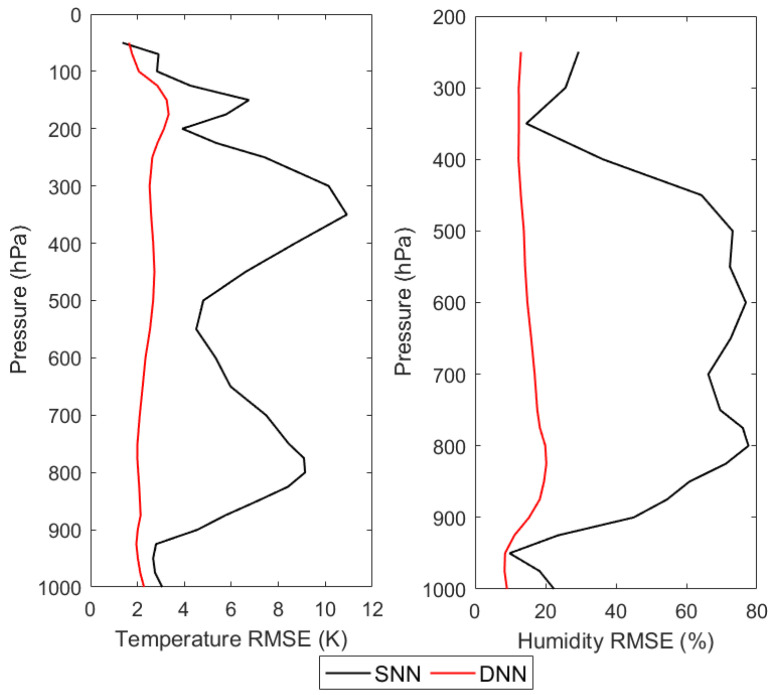
The retrieval RMSEs of the NN-based retrievals using the simulations with respect to ERA-Interim.

According to the experimental design of the third retrieval scheme, the DNN-based corrected brightness temperature and the SNN-based corrected brightness temperature obtained in the first retrieval scheme are input to the DNN-based and SNN-based retrieval models using the simulations, respectively, to obtain the retrieval results of the atmospheric temperature and humidity profiles, and the comparison of the retrieval accuracies is shown in Figure 11, and Table 6.

**Figure 12:**
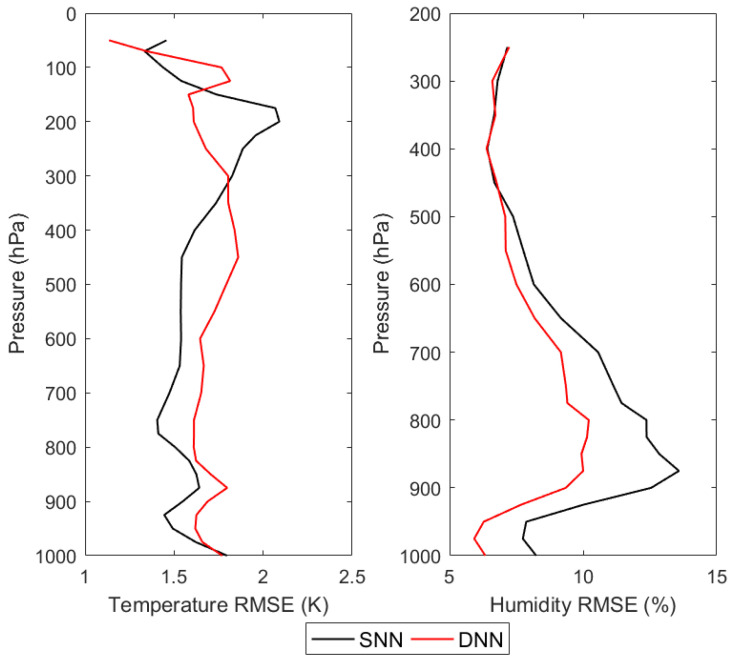
The DNN-based and the SNN-based retrieval RMSEs using MWHTS simulated brightness temperatures.

From Figure 11, it can be seen that, for the retrieval results of DNN, the retrieval accuracies of both temperature and humidity profiles are comparable to those of the first retrieval scheme and the second retrieval scheme. However, for the retrieval results of SNN, the retrieval accuracy of the temperature profile at 350 hPa is about 12 K and the retrieval accuracy of the humidity profile at 800 hPa is about 79%. Such poor retrieval accuracy undoubtedly proves that the application of SNN in the third retrieval scheme is a failure. In order to find the reason for the failure of SNN applied in the third retrieval scheme, another experiment is carried out in which the simulated brightness temperature in the testing dataset is used to replace the corrected brightness temperature used in the third retrieval scheme, which is input to the DNN-based and SNN-based retrieval models using the simulations, respectively, to obtain the retrievals of atmospheric temperature and humidity profiles. The retrieval accuracies are shown in Figure 12 and Table 7.

From Figure 12, it can be found that for the temperature retrieval results of SNN, the retrieval accuracy of SNN is improved substantially compared with that of the NN-based retrieval using the simulations, and comparable to that of DNN can be obtained, even slightly higher in the range of 300–1000 hPa. For the temperature retrieval results of DNN, the retrieval accuracy is improved by 0.8 K at 500 hPa compared with that of the NN-based retrieval using the simulations. For the humidity retrieval results of SNN, the retrieval accuracy of SNN is improved substantially compared with the retrieval results of the NN-based retrieval using the simulations, and the humidity retrieval accuracy of DNN is improved by 8.2% at most. It is obvious that both neural networks obtain higher retrieval accuracy compared to that of the NN-based retrieval using the simulations. The reason for this is that the neural networks are trained to establish a statistical relationship between the simulated brightness temperature and the atmospheric temperature and humidity profiles, which is relatively simple compared to the statistical relationship between the corrected brightness temperature or the observed brightness temperature and the atmospheric temperature and humidity profiles in the NN-based retrieval using the simulations, because there is no observation bias. However, when the corrected brightness temperature is input to SNN for retrieval in the NN-based retrieval using the simulations, the retrieval fails as shown in Figure 11, which is due to the poor generalization ability of SNN itself. Therefore, for the NN-based retrieval using the simulations, SNN fails in the retrieval, while DNN outperforms due to its stronger generalization ability.

### 4.3. Algorithm Stability Test

**Figure 13:**
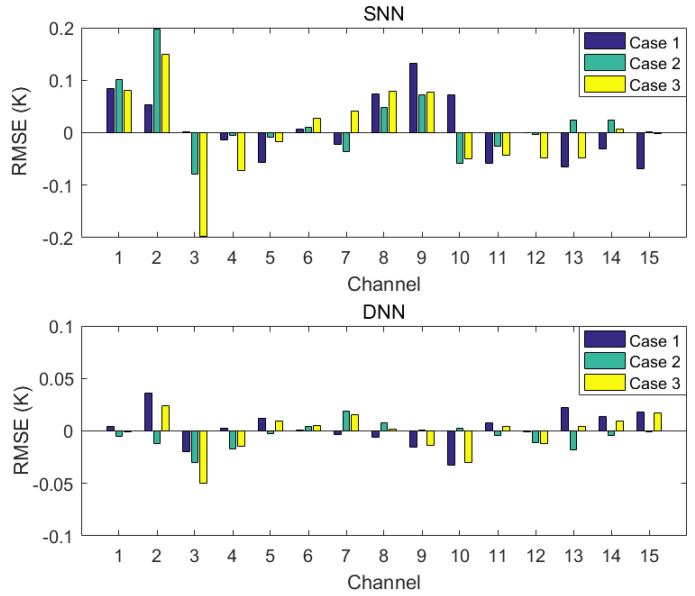
The stability test result of SNN and DNN in MWHTS-observation-bias correction.

**Figure 14:**
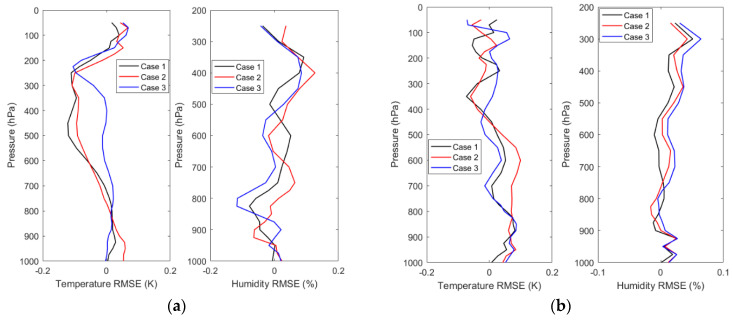
The stability test results of the retrieval of atmospheric temperature and humidity based on SNN and DNN using MWHTS observations: (**a**) SNN and (**b**) DNN.

The stability test of the algorithm is required when the neural network is applied to retrieve the atmospheric parameters using passive microwave measurements. The weights and biases are set randomly at the start of the training of the neural network. The performance of a well-constructed neural network for atmospheric parameter retrieval is not affected by the different weights and biases used at the start of training. Therefore, the stability testing experiments of DNN and SNN in the three retrieval schemes are carried out, simultaneously. Taking the 1DVAR retrieval and the NN-based retrieval using the observations as examples, one must retrain the DNN and SNN models with randomly initialized weights and biased for three separate times, respectively. Then they are applied to the same MWHTS observations as those of the 1DVAR retrieval and the NN-based retrieval using the observations. The differences in the RMSE of bias correction between the three bias corrections and the bias correction in Section 4.1 are shown in Figure 13. The differences in the retrieval accuracies between the three retrievals and the retrieval in Section 4.2.2 are shown in Figure 14.

For the bias-correction results of SNN and DNN for MWHTS observation bias, it can be seen from Figure 13 that when different weights and biases are used at the start of training of SNN and DNN, the differences of the RMSEs of bias corrections of SNN-based bias-correction model between three bias corrections and the bias correction in Section 4.1 stay within 0.2 K for all 15 channels, while those of DNN-based bias-correction model remain within 0.05 K in all 15 channels. Although the bias-correction difference of 0.2 K due to SNN can be negligible to the atmospheric temperature and humidity results, DNN shows more stability when applied to the MWHTS-observation-bias correction compared to SNN. For the retrieval results of SNN and DNN using MWHTS observations, it can be seen from Figure 14 that three separate trainings used different weights and biases at the start of the training of SNN and DNN lead to comparable differences in the retrieval results of the temperature profiles, which are both kept within 0.2 K. While the difference in the humidity profiles retrieval is smaller for DNN. In summary, SNN and DNN have good and comparable stability when applied to retrieve temperature profiles using MWHTS observations, and SNN and DNN also have good stability when applied to retrieve humidity profiles using MWHTS observations, but DNN performs more stable.

In conclusion, both SNN and DNN can be successfully applied to the 1DVAR retrieval and the NN-based retrieval using the observations, and DNN shows better performance than SNN in both MWHTS-observation-bias correction and the retrieval of temperature and humidity profiles using MWHTS observations. However, in the NN-based retrieval using the observations, the application of SNN fails, while DNN achieves a successful retrieval based on MWHTS simulations due to its stronger generalization ability. For the three retrieval schemes, although the 1DVAR retrieval and the NN-based retrieval using the observations obtain comparable retrieval accuracies of atmospheric temperature and humidity profiles, they have their advantages and disadvantages. Although the 1DVAR algorithm does not require a large amount of historical data for learning the sample features, the parameters of the physical retrieval system need to be set before retrieving to ensure a high retrieval accuracy, which is computationally intensive and expensive. In the NN-based retrieval using the observations, although the retrieval operation is simple, it is necessary to establish representative sample data for the training of NNs to ensure high retrieval accuracy when applied to new observed brightness temperature. For the NN-based retrieval using the simulations, although the retrieval accuracy is slightly poorer compared with the former two retrieval schemes, it has the advantage that the data-matching errors between satellite observations and atmospheric parameters can be disregarded because the atmospheric parameters can be directly used to generate simulated brightness temperatures, which can satisfy the demand of the neural network for a large number of data samples, and it is easy to establish a representative sample dataset.

## 5. Conclusions

In this paper, the application of DNN in the retrieval of atmospheric temperature and humidity profiles from FY-3D/MWHTS was investigated, mainly involving the application of DNN in MWHTS-observation-bias correction and in retrieving atmospheric temperature and humidity profiles using MWHTS observed brightness temperature or MWHTS simulated brightness temperature. In order to verify the performance of DNN applied in retrieving, a study on the application of SNN in the same situation was also carried out. It is found that, in the three retrieval schemes of atmospheric temperature and humidity profiles that use MWHTS measurements, DNN shows superior performance and better stability than SNN in both MWHTS-observation-bias correction and retrieval of atmospheric temperature and humidity profiles. Therefore, the results indicate that DNN and other deep learning algorithms have great potential in the applications of microwave remote sensing.

It is noted that the retrieval experiments of temperature and humidity profiles in this study were conducted under all-weather conditions and do not classify the MWHTS observations for different atmospheric states, such as clear sky, cloudy, and rainy. In practical applications, higher retrieval accuracy can be obtained if the brightness temperature classification according to different atmospheric states is realized based on the brightness temperature characteristics, and then the atmospheric temperature and humidity profile is retrieved under different atmospheric states. However, it is difficult to classify satellite observed brightness temperature under different atmospheric states based on the characteristics of satellite observations at present. The study of data classification of satellite microwave observation under different atmospheric states by deep learning algorithms to further improve the retrieval accuracy of atmospheric temperature and humidity profiles using satellite passive microwave observations is the focus of future work.

## Figures and Tables

**Table 1:** Channel characteristics of MWHTS.

Channel	Frequency (GHz)	Sensitivity (K)	In-Flight Sensitivity (K)	Calibration Accuracy (K)	Peak WF Height (hPa)
1	89.0	1.0	0.23	1.3	window
2	118.75 ± 0.08	3.6	1.62	2.0	30
3	118.75 ± 0.2	2.0	0.75	2.0	50
4	118.75 ± 0.3	1.6	0.59	2.0	100
5	118.75 ± 0.8	1.6	0.65	2.0	250
6	118.75 ± 1.1	1.6	0.52	2.0	350
7	118.75 ± 2.5	1.6	0.49	2.0	surface
8	118.75 ± 3.0	1.0	0.27	2.0	surface
9	118.75 ± 5.0	1.0	0.27	2.0	surface
10	150.0	1.0	0.34	1.3	window
11	183.31 ± 1.0	1.0	0.47	1.3	300
12	183.31 ± 1.8	1.0	0.34	1.3	400
13	183.31 ± 3.0	1.0	0.30	1.3	500
14	183.31 ± 4.5	1.0	0.22	1.3	700
15	183.31 ± 7.0	1.0	0.27	1.3	800

**Table 2:** The DNN configuration.

Retrieval Scheme	Number of Hidden Layers	Number of Neurons in Hidden Layer	Activation Function
1st	2	500,500	ReLU
2st	2	300,300	ReLU
3st	2	300,300	ReLU

**Table 3:** The SNN configuration.

Retrieval Scheme	Number of Hidden Layers	Number of Neurons in Hidden Layer	Activation Function
1st	1	33	ReLU
2st	1	21	ReLU
3st	1	24	ReLU

**Table 4:** Summary of the retrieval RMSE of the 1DVAR retrieval with respect to ERA-Interim.

	Temperature RMSE (K)			Humidity RMSE (%)		
Level (hPa)	SNN	DNN	Without Bias Correction	SNN	DNN	Without Bias Correction
100	0.51	0.61	1.85	-	-	-
300	1.85	1.52	3.86	8.00	6.49	8.20
500	2.01	1.69	3.19	17.94	11.01	18.75
800	1.58	1.49	2.48	27.12	24.05	37.93
950	2.11	2.09	3.54	12.01	11.97	20.01

**Table 5:** Summary of the retrieval RMSE of the NN-based retrievals using the observations with respect to ERA-Interim.

Temperature RMSE (K)			Humidity RMSE (%)	
Level (hPa)	SNN	DNN	SNN	DNN
100	1.85	1.82	-	-
300	2.31	2.21	10.94	10.39
500	1.95	1.86	12.63	11.90
800	1.94	1.69	18.42	16.81
950	1.81	1.68	8.26	7.63

**Table 6:** Summary of the retrieval RMSEs of the NN-based retrievals using the simulations with respect to ERA-Interim.

Temperature RMSE (K)			Humidity RMSE (%)	
Level (hPa)	SNN	DNN	SNN	DNN
100	2.89	2.05	-	-
300	10.13	2.51	25.67	12.40
500	4.80	2.66	73.11	13.85
800	9.13	2.03	78.78	18.40
950	2.67	2.01	9.79	8.52

**Table 7:** Summary of the DNN-based and the SNN-based retrieval RMSEs using MWHTS simulated brightness temperatures.

Temperature RMSE (K)			Humidity RMSE (%)	
Level (hPa)	SNN	DNN	SNN	DNN
100	1.44	1.76	-	-
300	1.83	1.80	6.80	6.60
500	1.54	1.79	7.38	7.08
800	1.50	1.61	12.37	10.21
950	1.49	1.61	7.87	6.27

## Data Availability

Not applicable.
